# Should the Nurse Articulate?

**Published:** 1918-12-21

**Authors:** 


*To the Editor of* THE HOSPITAL.

### Should the Nurse Articulate?

*To the Editor of* THE HOSPITAL.

SIR,—I have read with keen interest the letters of "Ierne" and "Merely a Staff Nurse in the Northern Command," and endorse all that has been said and suggested. They have voiced the feeling of many of their fellow-nurses. As suggested by the latter, nursing is considered the noblest of professions, and one is forced to admit, especially after four years of war, the asset the fully-trained nurse has been to the nation. The true nurse enters hospital with the highest and best motives, realising the former. Nurses, however, are still "human," and require the necessities of life as others. They cannot live on "sentiment" and "paper talk." The former writer spoke of a letter received from the Director-General. In our Command a similar letter was received.

It is time someone took up our cause in a practical way and achieved for us what justice demands. We give the best of our lives, the work is not easy, and yet ou rsalaries are on the lowest scale.

"*Re* the gratuity, we certainly understand it is paid for "completed service," but why should it be forfeited if a nurse leaves the Service on good grounds? For example, the T.F.N.S. Standing Orders state: "If she has relinquished her employment for reasons *not satisfactory* to the Army Council she will forfeit her title to the gratuity." What is meant by "not satisfactory"? I have lately met nurses who married during the war, and since hostilities ceased have wished to leave the Service to prepare for the return of their husbands. There is no distinction made between married and single, and hence their only course is to resign. So after serving for three or four years they are told they must forfeit their gratuity. Evidently "marriage is one of the unfavourable things"—at least, it appears so. Is this condition of things fair after faithful service? Most people who have been in "war service" are being considered under the former circumstances. Why not the trained nurse?

We look to the College of Nursing to act for us, and if they fail to respond we must seek another way. Now is the opportune time, and if we fail to stand now we can never hope for a better, to secure the standing and consideration worthy of our profession.

"ANOTHER MERE STAFF NURSE."

Glamorgan, December 11, 1918.

